# Infant Feeding and Infant Foods

**Published:** 1882-05

**Authors:** 


### Selections.

### INFANT FEEDING AND INFANT FOODS.

MR. VICE-PRESIDENT, LADIES AND GENTLEMEN, MEMBERS AND DELEGATES OF THE MEDICAL SOCIETY OF THE STATE OF NEW YORK: I hold in my hand a report of the committee appointed to co-operate with the New York Society for the Prevention of Cruelty to Children, "in all things pertaining to the physical and moral safety of infants and children." The report I refer to was read in last year's meeting, and has been widely distributed by the above-named society. One of the measures contemplated and advised in it was, that there should be a place or places in large cities, where the infants and young children of the poor might be supplied, at cost, with the simple though sufficient articles of food. These simple and sufficient articles of food were milk, barley, oat-meal, and, for those no longer infants proper, eggs. Their value as infant food for those who were without breast-milk, was taken for granted, and considered almost in the light of an axiom. To make it so has been one of the duties of my life. Truth, however, is frequently suspected because of its very simplicity, and often do we see complicated and roundabout means and measures resorted to in preference to plain and direct ones. Now, what I have to say may, in part, appear very common-place and trifling, because of its application to common-place things and interests. But questions are involved which it has taken, and will still take much time and labor to solve. To render the results hitherto obtained available, the normal feeding of the infants possible, and to protect them from the injuries inflicted by ill-directed love, ignorance, and the evil-devised plans and frauds of what is called commerce and industry, will be the object of the brief remarks which I shall have the honor to make before you in regard to the dangers to which our infants are constantly exposed.

Mortality amongst infants is very great. In Europe, out of one hundred born alive, eighteen die before they reach the end of their first year. Out of one hundred who die at all ages and of all diseases, in New York City, twenty-eight are less than a year old. The causes of these deaths vary; it is a characteristic fact, however, that of all those taking place in infants under a year, from 40 to 53 per cent. are the results of diseases of the organs of digestion.

Thus, the attention of the physician called to see a sick child is mostly claimed by the alimentary canal. His care and that of the mother or attendant is therefore due to preventive measures mostly. The best preventive measure, and sometimes it proves curative, is the breast-milk of mother or wet-nurse. That is an axiom, an indisputable law of nature, as long as the circumstances of the case are favorable. In view of the great mortality in the first two months, breast-milk is the one and indispensible food for those under two months. A mother who refuses to nurse *at least that time* for other than reasons of health or life, is an accomplice in causing, perhaps even the only cause of, the death of her offspring. It is true that a baby may be taken sick with intestinal disease in spite of being nursed at the breast, for there are many causes of disease; it may, however, occur that babies are taken sick because of being at the breast. And it is those cases that both mothers and physicians ought to be well acquainted with. Sometimes it is not the breast-milk which is at fault, in the beginning, but the faulty use it is put to. Many babies suffer intensely because they are not limited to intervals of from 2 to 4 or 5 hours, as required by either age or constitution. In their cases, by too frequent feeding, both the milk of the mother and the digestion of the infant, are impaired. Here regularity is the sole indication. Sometimes, though fortunately in few cases only, there appears to exist an idiosyncrosy not explained, on the part of the baby who cannot thrive on the milk of the mother, and may do so after a change of food. In many cases, however, there are demonstrable dangers in the very breast-milk of either mother or nurse; there may be an undue percentage of fat, or of cheese, or of salts, or of sugar, or even accidental admixtures. These may occur in the secreting organ (thus blood may be found in the milk) or be traceable to the circulating blood of the whole system; of the latter they may be the very constituents, or foreign bodies floating in it. They can be classed as either morbid dispositions or as actual admixtures. Women suffering from constitutional syphilis, chronic consumption, or anæmia, extensive rachitis, severe nervous derangement, hysterical or other, those suffering from care or hard work, and those who are compelled to take a great deal of medicine, will serve their babies best by not nursing them at all.

In regard to the influence of the medicines, the opinions have been divided. It was claimed that milk being a secretion of the gland, and not a transudation from the blood, would not contain foreign material to any great extent. That is true so far as an absolutely healthy woman and normal milk are concerned. But the first period of lactation yields colostrum, not normal milk, and very often the latter is changing into a colostral condition, such as it was soon after birth, containing different-shaped fat globules, more sugar, soluble albumen, in fact, real blood serum. This may take place in every case of impaired health. And the more serum of blood is contained in any milk, the easier is the admixture of soluble substances circulating in the blood. Such substances are ethereal oils, coloring matter, iron, iodide of potassium, arsenic, zinc, mercury, salines, bismuth, lead, antimony, nitrate of potassa, magnesia, all of which have been frequently found in the milk, and frequently missed. They will be missed the more frequently the healthier the woman and its mammary secretion. But, as I formulated the subject some years ago, milk secreted from an insufficient mamma, by a woman not in full health and vigor, by an old woman, by a very young woman, by an anæmic woman, by a convalescent woman, who has consumed a large portion of her albumen, be it circulating or tissue albumen, by a neurotic woman with frequent disturbances of the circulation—milk, in fact, which is not the normal transformation of the elements of the mammary glands, but consists of more or less transuded serum, is apt to be impregnated with elements circulating in the blood. The indications on the one hand for permission to nurse, on the other, for the administration of medicines to a nursing woman, require, therefore, a greater strictness than is usually conceded. At all events, the good results obtained in many cases of ailment on the part of the infant, by artificial feeding, in preference to nursing, are better than accidental.

Those infants who are thus deprived of breast-milk, or never had it, or have an insufficient supply of it, require artificial feeding. The material resorted to ought to be as much like mother's milk as possible; and naturally, when human milk cannot be had, animal milks are selected. Amongst them, only two are available to any extent, that of the goat and of the cow. If there be any objection to either, it is principally valid in regard to the former. For the chemical incongruities and other difficulties, to which I shall briefly allude in regard to cow's milk, are even more pronounced in the case of goat's milk.

My remarks of this evening I expect to be of a practical nature throughout, and brief. Thus I shall not try to go into chemical or physiological questions beyond the time allotted or the opportunity afforded. Therefore, I shall allude to but two points which it is interesting to appreciate, in order to understand why it is that cow's milk, as a sufficient substitute for mother's milk, must necessarily be a failure.

Human milk and cow's milk differ mainly in that the percentages, and probably also the properties, of fat and of caseine contained in them, differ widely. The percentage of fat in cow's milk is larger than in human milk, and, on close observation, we shall learn that woman's milk contains as much fat as the young digestive organ can tolerate. From what we know of the diastatic power of the infant pancreatic gland, we must conclude that its property of digesting fat is but slimly developed soon after birth; and daily observation proves that such is the ease. When you examine chemically the normal alvine digestions of the infant, well mixed, uniform, and yellow, you will discover not only fat acids, but free fat to the extent of about 12 per cent. Some more fat leaves the intestines in a saponified condition. Many of the passages which have been believed to contain caseine in excess, because of their white color, were found to contain large quantities of fat. And all this happens when the normal infant is fed exclusively on normal breast-milk. Now if much fat is not required nor absorbed under normal circumstances, if fat acids form so easily when the digestion is apparently normal, with the constant tendency of deranging digestion; if we know, as we do, that even normal breast-milk contains more fat than the infant has any use for, we must admit that in artificial feeding, with cow's milk or other material, we are in greater danger from giving too much fat than too little. Good milk has been called that which contains most fat, say from four to five per cent. The more cow's milk deserves the title of being good—that is, fat—the greater is the danger arising from giving it to infants without considerable modification. This result authors appear to gradually appreciate. When Forster fed a baby of four months with cow's milk and rice decoction in the proportion of 1 to 4, all the albuminous substances and sugar of this mixture had disappeared when the fæces came to be examined. Thus they were entirely digested, but of the ashes 34, and of the fat 30 to 40 per cent. were found unchanged. This experiment proves, in the opinion of Prof. Bollinger, whom I rejoice in quoting, that a milk less rich in fat is more adapted to serve as infant food than one with a large percentage of fat; and further, that cows whose milk is destined to be used as infant food, ought to be fed so as to reduce rather than increase the amount of fat contained in it. A good authority, Prof. Feser, makes the distinct statement that the milk he examined on the Alps of Switzerland, and which he prefers greatly as children's food, contained less fat than that from the cows in the neighborhood of a large city. If that be so, and it is so, I recommend as a matter of practice that the morning milk, with its lesser percentage of fat (butter), should be given in preference to evening milk, if any at all.

Now, if the excess of fat be one of the causes why cow's milk should not be employed as infant food, why not remove it or diminish its percentage? Easily said, not easily done; for when milk is allowed to stand, and the cream to rise, the milk at the same time changes its reaction. Instead of being alkaline or neutral, it becomes acid, and acid milk is a danger which an infant must be protected from. Some years ago, Lefeldt and Delaval invented each an apparatus which, by rapid centrifugal motion, separates the cream from the milk very thoroughly. Such an apparatus may, perhaps, be found useful in reducing the normal fat of cow's milk to an acceptable standard in the future. For the present, however, I know of no proceeding which accomplishes the end in view; and, while submitting to the unavoidable, I must protest against the avoidable occurrence which is reported to have taken place in a court of justice but lately. In one of the skim-milk police cases—and unfortunately there are less "cases" than adulterations—the expert for the defense, a well-known chemist, tried to clear his client by proving him a public benefactor, inasmuch as Dr. Jacobi objected to an excess of butter in the milk used for children.

Caseine is represented in cow's milk in a larger percentage than in human milk. To reduce its relative quantity, compared with the rest of the solid constituents, is possible by the increase of these only. There is no method by which its absolute quantity in a given amount of milk could be diminished, except such as would render the remaining part of the milk unfit for use. This diminution, moreover, would have to be a very thorough one, for a mixture containing more than 1 per cent. of caseine has been found to be indigestible by Hammarsten, and by Biedert. But these are not the only objections to cow's milk caseine. Not only is its percentage in the milk too great, its chemical and physical properties differ greatly from that contained in human milk. Mineral, acetic, and tartaric acids, epsom salts, phosphate of lime, form a dense and hard cogulation with the caseine of cow's milk, while human milk congulates in loose and small flakes only, under the same circumstances. Cow's' milk, when introduced into the infant stomach, forms hard curds; if the organ be strong, or the infant liable to vomit easily, as most of them are, thick massive pieces of cheese are thrown up, and relief will follow. If no vomiting occur, these hard masses are propelled into the intestinal tract, where sometimes they give rise to obstruction. Sometimes, however, they irritate the canal to such an extent, that diarrhœa is the result. In both cases lumpy cheese is the main constituent of the evacuations. In every case of infant diarrhœa, the examination of the passages might be made; in many cases the appearances will be found as described, and no case of the kind will terminate favorably unless the food be changed. To say that these cases will occur, would not do justice to the facts. They are very frequent, I have seen hundreds; but lately a child of sixteen months of age, and of six pounds in weight, was presented to me with the same symptoms, and with the additional remark, not only that everything had been done by the medical adviser to stop the exhausting diarrhœa, but also, that a cow had been purchased for the sole purpose of furnishing pure milk for the suffering baby. That suffering baby was instantly improved when the pure milk was stopped, and more suitable food was substituted.

The objections to cow's milk, as the exclusive diet of infants, in place of mother's milk, which cannot be had, are dependent on its very chemical composition and physical properties. These cannot be changed by the most studious and successful attempts at procuring the most uniform and pure article possible. That, however, does not prove that a bad or indifferent milk is as good and useful as the very best milk from the very best cows. The inference is rather this: that the employment of inferior milk is criminal when good milk can be had through precaution, care and industry. To have accomplished that purpose is the pride of several European cities. In some it was through medical societies that the idea was started and put into effect, to procure as good milk as cows of good stock, well fed under favorable sanitary conditions, could furnish. Stuttgart had the first establishment of the kind; that in Frankfort-on-the-Main commenced its operations in 1877, with thirty cows, and increased that number to eighty-one in 1881. The amount of milk produced and delivered to consumers in 1881 was 313,563 litres (quarts), more than ten quarts per cow daily. The establishment is private property, but to earn the support of the public, and to secure the desired coöperation of the physicians of the town, the proprietor bound himself not to engage in any other business, and solicited the control and advice of a committee, consisting of three delegates of the medical society, one veterinary surgeon and one chemist. Great pains are taken in the purchase of animals of a good stock, all of them thus far being imported from Switzerland. Before they are admitted they are examined by the veterinary surgeon, who superintends the institution, and keeps a regular record of those who may fall sick. Two months before calving the milk is not sold at all. The age of the cows of from three to eight years is considered the only available one. The stables are well ventilated, and washed, drained and connected with the system of city sewers. The floors of the stalls, the ceilings and the lower part of the walls are cemented. The skin of the animal is looked after with great care. The stables are so near the city limits that the milk cannot suffer by transportation. It is tightly corked in half-quart and quart bottles (this process being cautiously watched), and speedily delivered. Printed directions for its further treatment accompany the article. Besides, stress is laid on avoiding a prejudice on the part of the public, to which I have raised objection long ago, with but very partial success, viz.; not to select the milk of a special cow for the special use of the same individual, but to mix and equally distribute the milk of the whole dairy.

A great innovation for practical purposes, based on good experiments and long experience, is that no change of food takes place, and poisonous, purgative or otherwise injurious admixtures to the food of the animals are avoided. Dry food only is given, viz., 15 kilog. (30 pounds) of hay, 6½ kilog. (13 pounds) of meal and bran, 6 grammes (1½ drachms) of salt, and spring water to drink.*

Human knowledge and foresight have almost exhausted themselves in these praiseworthy efforts. But the result of them all is good cow's milk, and no equivalent for mother's milk. But do I mean to say that cow's milk, given pure, or cow's milk mixed with water, is absolutely injurious, poisonous, to babies? Far from it. The undeniable fact that there are infants who thrive on that exclusive diet, or whose general condition of health remains such that no medical or medicinal interference in their behalf appears called for, would give the lie to such an exaggerated opinion. Still, there are a great many infants who appear to thrive better than they actually do. Many grow fat and

---

* Dr. V. Cnyrim, in "Frankfort-on-the-Main, Its Hygienic Conditions and Institutions." Ed. by Dr. A. Spiess, 1881.

rotund on improper food, no immediate harm makes its appearance, and still you see many such as increase in weight, even more than the average infant, and still lay the gradual foundation to future ailing. For such fatness and rotundity means, too frequently, rachitis, and requires watching and change of food. Still, the number of infants remaining in good condition on that food is not very small. What does it prove? Nothing else but that the digestive processes permit of a certain latitude, that nature does not do routine work, and that the sum total of vital processes do not respond to certain occurrences, or influences, like reagents in a chemical test, where the same process always yields the same results. There is no food on which certain infants will not thrive. But when, in a larger percentage of cases, the same unfortunate results are exhibited, and when these can be traced to their exact and uniform cause, that particular food is to be condemned. The assertion of many that cow's milk is an exact substitute for human milk, is the counterpart of that which claimed equality for animal and human blood. The practice, based upon this assumption, resulting, as it did, in the transfusion of animal blood into the human circulation, outlived itself very speedily.

Cow's milk, as a universal substitute for mother's milk, has lost its credit with many through the differences in the article; which, however, as long as no adulterations are perpetrated, are less marked than the same secretion of the human being. If that were not so, how does it happen that all over the civilized world substitutes are sought for, offered and purchased, though milk be as cheap and handy as anything else? Why is it that to avoid cow's milk untold risks are run in procuring more expensive, more unknown and more unreliable vegetable compositions, which seldom keep the promises loudly displayed on the labels? What are the promises? What does it mean when they claim to take the place of mother's milk?

Mother's milk is considered the best prepared article of food known, though, as you have been told before, it may be—it could have been improved upon. Perhaps it is the best, though; at least there was a time when we were all of that opinion; for perhaps the surplus fat, which passes off undigested, may serve some purpose hitherto unknown. An ideal article of food must serve two purposes and consist of two classes of constituents. It must, in the infant, supply the growing tissue with material sufficient to take the place of that which is constantly wasted, and to allow a surplus for increase; and, secondly, supply fuel for the purpose of keeping up the production of an equable temperatu.e and the functions of the organs, mainly those of respiration. The first requirement is fulfilled by the proteinous, or albuminous, substances, the other by carbo-hydrates. This statement would require modification, if it were the object to be absolutely correct, for the two classes will supply each other, act vicariously for each other, even change into each other, under certain circumstances, in the complex machinery of the human system. The albuminous tissue-builder in the milk is mainly caseine, sometimes, also, albumen; the second class is principally represented by fat and sugar. In vegetables the first class is represented in the gluten, the second mainly in the starch. If you add that in the milk, the ideal food, the proportion of the first class to the second is about 1 to 4, the vegetable substitutes are to be judged, in regard to their mere chemical composition, to that formula of about 1 to 4.

But it is not the chemical formula alone which determines the rank of a substance as a nutriment. To the equivalents of the chemical formula of cow caseine infant mathematics would not object, did not the infant stomach revolt against it. Thus it is not exclusively the chemistry of an article, but its digestibility, which comes into question. Now, not everything is equally digestible for everybody, sick or well, old or young, adult or child, child or infant, infant or newly born. And particularly is that so with regard to starch, which forms such an overwhelming part in the composition of vegetables, and particularly of those which are mainly used for the purpose of the manufacture of infants' food.

Starch is changed into sugar and rendered digestible by the secretion of two sets of glands, the salivary glands and the pancreas. The latter does not does not possess that function before the end of the first month of life. The former begins to perform the same function, to a certain degree, immediately after birth.

The experiment has been made by Schiffer, Korowin and many others. Starch, inclosed in a small bag, is introduced into the mouth of the infant. After a certain time the starch is found to be transformed into sugar. To collect fifteen grains of saliva in a baby of two or three weeks took from fifteen to thirty minutes; in a baby of two months, one or two minutes. Thus two facts result from these experimental observations: 1. That starch is digestible at any age. 2. That the transformable and digestible amount of starch is but very small in the first few weeks of an infant, and increases gradually. About the eleventh month the digestive property of the saliva is equal to that of an adult, when the quantities are equal.

The inferences to be drawn are: 1. That farinaceous foods may contain starch, but starch must not be the main part of the food. 2. That when they contain a great deal of starch it is best to change it, by a thorough baking process, into dextrine, which is changeable into sugar, and is easily absorbed and utilized. 3. That those farinacea ought to be selected for infant food in which the proportion of gluten to starch favors the former.

There is another consideration of vast importance, which, when the infant foods now in the markets were first manufactured, could not be known. Starch is not the same article everywhere. In different substances it requires different times to be transformed into sugar. Potato starch requires 2 to 4 hours, that of peas $1\frac{3}{4}$ to 2 hours, wheat $\frac{1}{2}$ to 1 hour, barley 10 to 15 minutes, oats 5 to 7 minutes, rye 3 to 6 minutes, maize 2 to 3 minutes.

Guided by these facts, I invite you to scan with me a number of farinaceous substitutes—their claims, promises and powers. You will notice, there is not one of the men or companies who supply them but is loud in the praise of his gift to infancy; not one but ranks in his actual or alleged estimation as the arch-benefactor of mankind.

Justus von Liebig's (the great chemist) substitute consisted of the flour of wheat and barley malt, bicarbonate of potassium, water and milk in certain proportions. For about twenty years this preparation has been the subject of eulogies and reproaches, until in its original form it has been nearly given up; for its home preparation is not so easy as its inventor claimed.

It requires too much care and attention from a person of intellectual undersize. Besides, everything containing sugar is liable to decompose in summer by fermentation and moulding; all preparations really dry have a bad taste. Moreover, the phosphate of lime, which, it is true, is found in sufficient quantity in both wheat and malt, does not get into the solution, and finally, for its preparation you always require milk—milk in winter and summer, milk in city and country, with all its actual or alleged dangers. Still more, it has, though prepared ever so carefully, proven unsuccessful in too many cases. Diarrhœa has often resulted from its use. Numerous attempts have been made to put it up for the market in such a shape that little time and no intellect might be required for its final use. Loefflund's article has the merit of being no fraud, as far as I know; that of Liebe I paid attention to when it was first largely advertised for sale. A dozen bottles contained in a box were examined; their consistency and composition differed considerably. Another preparation, alleged to be compounded according to the prescription of the great chemist, is sold with the name of his son, Herman von Liebig, attached to the recommendation of the label. He has a great deal too much to say in its praise. "1. This remarkable preparation accomplishes everything that can be expected of a substitute. 2. Although the flour is a perfect nutriment in itself, I still recommend on my labels its mixture with cow's milk. 3. Where good cow's milk cannot be had, condensed milk may take its place. 4. Infants fed on cow's or condensed milk ought to have it mixed with my flour." You see the unsophisticated public must come to the conclusion that the object and destination of this food is no other but to be purchased.

Next in order is Nestle, as widely known as Plantation Bitters and cider vinegar. This food consists of milk, wheat flour (alleged to be baked, for the purpose of crushing the cellulose and cell membranes), sugar and salts. As a chemical critic says (H. Mueller, in *Pharm. Centralhalle*, xvi., 1875, n. 34), he publishes no analysis, but advertisements only; and he knew how to advertise. I remember well when the people of New York awoke one morning to find their libraries increased by a big pamphlet containing the new revelation. It was written by no less a person than an emeritus university professor of pathology, in his time a justly celebrated man, Lebert, who passed his very old age in the neighborhood of where Nestle had established his health, life and money-breeding factory. Every such pamphlet commences with a declaration of principles. This begins by telling the reader that cow's milk is more "nourishing" than human milk; that, however, the addition of water does not make it any more similar to the latter; and that the nursling ought to have cow's milk at once alongside that of his mother—probably to improve upon nature's plan. Then why does he want or recommend substitutes, if cow's milk does so well—even better than well? For the simple reason that if people would feed their own cow's milk they would not purchase Nestle's; and in order to induce them to do the latter he tells you that your cows may be tubercular, may be fed improperly, their milk may be adulterated with water, or may contain disease germs. Nestle's cows are not tubercular, we are to infer, are not fed improperly, their milk cannot be adulterated, nor does it contain disease germs—indeed not. For it is the milk of *Swiss cows*, with such a halo round their horns that even that public-spirited institution called St. John's Guild, of New York, publishes an advertisement of Anglo-Swiss food on the last page of its pronunciamento on the bringing up and feeding of little children, which is distributed gratuitously, or for an annual contribution of three dollars.

[TO BE CONCLUDED IN JUNE NUMBER.]

